# The impact of sleep deprivation on cognitive function in healthy adults: insights from auditory P300 and reaction time analysis

**DOI:** 10.3389/fnins.2025.1559969

**Published:** 2025-04-09

**Authors:** Zhongkai Ren, Xiang Mao, Ziyue Zhang, Wei Wang

**Affiliations:** 1 Department of Otorhinolaryngology-Head and Neck Surgery, Tianjin First Central Hospital, Tianjin, China; 2 Institute of Otolaryngology of Tianjin, Tianjin, China; 3 Key Laboratory of Auditory Speech and Balance Medicine, Tianjin, China; 4 Key Medical Discipline of Tianjin (Otolaryngology), Tianjin, China; 5 Otolaryngology Clinical Quality Control Centre, Tianjin, China; 6 First Central Clinical College, Tianjin Medical University, Tianjin, China

**Keywords:** sleep deprivation, EEG, event-related potentials, P300, cognition

## Abstract

**Objective:**

This study aims to explore the effects of sleep deprivation on cognitive function in healthy adults, using auditory P300 event-related potentials and subjective reaction time as key assessment metrics.

**Methods:**

High-density electroencephalography (EEG) and the oddball paradigm were utilized to collect P300 event-related potentials (ERPs) before and after the sleep deprivation intervention, with a record of subjective reaction time. Participants were classified into acute sleep deprivation group and chronic sleep deprivation group based on the duration of their night shift history.

**Results:**

Following sleep deprivation, a significant prolongation in P300 latency and reaction time was observed among 26 subjects (*P* < 0.05). Specifically, the reaction time in the acute group increased significantly by 83.69 ms after sleep deprivation (*P* < 0.05). In contrast, the reaction time in the chronic group exhibited only a minor increase of 6.54 ms. Furthermore, a significant interaction effect between the duration of night shift history and sleep condition on reaction time was identified [*F*_(*a*X*b*)_ = 4.736, *P* = 0.040, η_*p*_^2^ = 0.165], suggesting that the influence of sleep deprivation on reaction time varies between the chronic and acute groups.

**Conclusion:**

Sleep deprivation induces cognitive impairment, with the acute sleep deprivation group experiencing more severe deficits. In contrast, the chronic sleep deprivation group demonstrated milder but chronic cognitive impairment.

## 1 Introduction

Sleep deprivation occurs when an individual is unable to meet standard sleep requirements due to environmental or personal factors. Sleep deprivation selectively impairs attention networks, primarily impairing brain executive function, followed by alertness (Dong et al., 2024). According to the revised third edition of the International Classification of Sleep Disorders (ICSD-3-TR) (American Academy of Sleep Medicine, 2023), sleep insufficiency syndrome is the clinical term for sleep deprivation. When a patient frequently experiences an irresistible urge to sleep or falls asleep during the day, they may be diagnosed with some form of hypersomnia, including sleep deprivation. Sleep deprivation can be categorized into acute total sleep deprivation (TSD) and chronic partial sleep restriction based on the quality and duration of sleep. Our study divided subjects into acute and chronic sleep deprivation groups based on the duration of night shift history.

A cross-sectional study revealed that around 29% of American adults suffer from sleep disorder-related issues, impacting the daytime work efficiency of an estimated 50-70 million individuals, with a more pronounced impact on women than men (Chapman et al., 2012). The incidence of sleep deprivation is higher in environments characterized by longer working hours, shift work, and high job stress (Luckhaupt et al., 2010). The effects of sleep deprivation on cognitive function show up in different ways; for example, executive functions (Aidman et al., 2018), attention performance (Stepan et al., 2020) and long-term memory (Ratcliff and Van Dongen, 2018), etc. Song et al. (2023a) have found that TSD damages visual search behavior and selectively impairs the earlier sub-stages of motor preparation. Sleep deprivation decreased the accuracy of the visual search task while increasing the reaction time variance. This suggested that participants manipulated the visual search task less accurately and with less stability after sleep deprivation. Sleep deprivation has a similar effect on auditory cognitive function. Chen et al. (2024), employing the two-back pronunciation working memory task, found that sleep deprivation not only impairs cognitive function but also triggers a compensatory mechanism that maintains the working memory performance. By affecting the allocation of attention resources, sleep deprivation may lead to a decrease in the efficiency of processing auditory information, which is manifested by prolonged reaction time and decreased accuracy in auditory tasks. Previous studies have focused on auditory cognitive impairment (Xu et al., 2022), possibly because sleep deprivation has more obvious effects on auditory attention and cognitive load (Yin et al., 2023). Moreover, the role of auditory processing in the brain is more complex, involving multiple cognitive layers of language comprehension and sound perception, and thus better able to demonstrate the multifaceted effects of sleep deprivation (Stothart and Kazanina, 2016). Comprehensive analysis indicates that sleep deprivation significantly impacts work efficiency, life quality, and physical health. Long-term sleep-deprived populations face an increased risk of mortality (Cappuccio et al., 2010).

Sufficient sleep is crucial for energy restoration and the storage of memory. Sleep deprivation or inadequate sleep can impair memory, judgment, discrimination, reaction time, and executive functions (Ray et al., 2012). Cognitive functions encompass various dimensions, such as perception, attention, memory, thinking, language, and executive functions. Dawson and Reid (1997) quantified the impact of sleep deprivation and alcohol on cognitive functions through comparison. Subjects who stayed awake for 24 h exhibited cognitive impairment equivalent to that caused by a blood alcohol concentration of 0.10%, meeting the threshold for mild alcohol intoxication. Long-term sleep deprivation has cumulative effects on adolescents’ cognitive function, significantly impairing their attention span and thinking ability (Fredriksen et al., 2004; Wolfson and Carskadon, 2003). Sleep deprivation exerts a significant negative impact on cognitive function comparable to alcohol intoxication, especially in school-age populations where long-term cumulative effects severely impair learning ability.

Various methods, such as questionnaires, functional magnetic resonance imaging, and EEG, can assess cognitive function. EEG reflects the bioelectrical activity of brain cells as the summed postsynaptic potentials of the synchronous activity of a large number of neurons. It is an overall representation of the electrophysiological activity of brain nerve cells on the surface of the cerebral cortex or scalp (Zhang et al., 2020). EEG offers exceptional temporal resolution, allowing for the capture of millisecond-level changes in the brain’s functional networks. This capability enables real-time monitoring of brain activity during cognitive tasks (Kingshott et al., 2000). By intentionally attributing specific psychological significance to stimuli, the electrical potentials elicited by various stimulus paradigms are called ERPs, reflecting neural electrophysiological changes during cognitive processes. The ERPs components most commonly used to reflect cognitive changes before and after sleep deprivation are N2, P2, and P3 (Kusztor et al., 2019; Zhang et al., 2019; Song et al., 2023b). N2 components reflect bottom-up processing and are associated with early conflict detection and error monitoring, primarily in the prefrontal region. The P2 component is linked to the early stages of active information processing and the selection of helpful information within the sensory cortex (Crowley and Colrain, 2004). The P3 component reflects top-down processing, including attentional resource allocation and cognitive control, mainly in the central parietal region (Salmi et al., 2019). The regulation of P3 components is one of the most critical reported findings in ERPs research on working memory updating (WMU). Song et al. (2022) examined the effects of 36-h total sleep deprivation (TSD) on visual WMU processes and found that TSD selectively altered P3 amplitude. Specifically, the P3 amplitude of the visual working memory updating task in the parietal region significantly increased after TSD, while P3 components in the frontal and central regions were undermined under TSD conditions. These findings indicate that TSD impaired cognitive functions in the frontal and central brain regions and triggered compensatory neural activity in the parietal brain region. The P3 potential is a well-established sensitive cognitive marker of sleep deprivation (Morris et al., 1992; Lee et al., 2003). The P300 component typically occurs around 280-550 ms after a stimulus and is characterized by a significant positive amplitude (Parker et al., 2021; Polich, 2007). The P300 reflects the brain’s cognitive processing abilities for auditory information, including information processing, response capability, attention, judgment, and working memory (Martins et al., 2011). P300 can serve as an objective indicator of cognitive functions. Therefore, this study selected P300 as an indicator to evaluate changes in cognitive function before and after sleep deprivation.

Although previous research has established the detrimental effects of sleep deprivation on cognitive function, there are still significant knowledge gaps in this area. Current studies have not investigated whether differences in cognitive impairment due to lack of sleep exist among individuals with different lengths of night shift history. Furthermore, most studies have relied solely on EEG technology without subjective verification, leading to a lack of consistency between subjective and objective analyses. Therefore, this study utilizes P300 combined with reaction time for a comprehensive analysis. Subjects are categorized into two groups based on their length of night shift history for comparative analysis, aiming to assess the impact of sleep deprivation on cognitive function from multiple perspectives.

## 2 Materials and methods

### 2.1 Subjects

**FIGURE 1:**
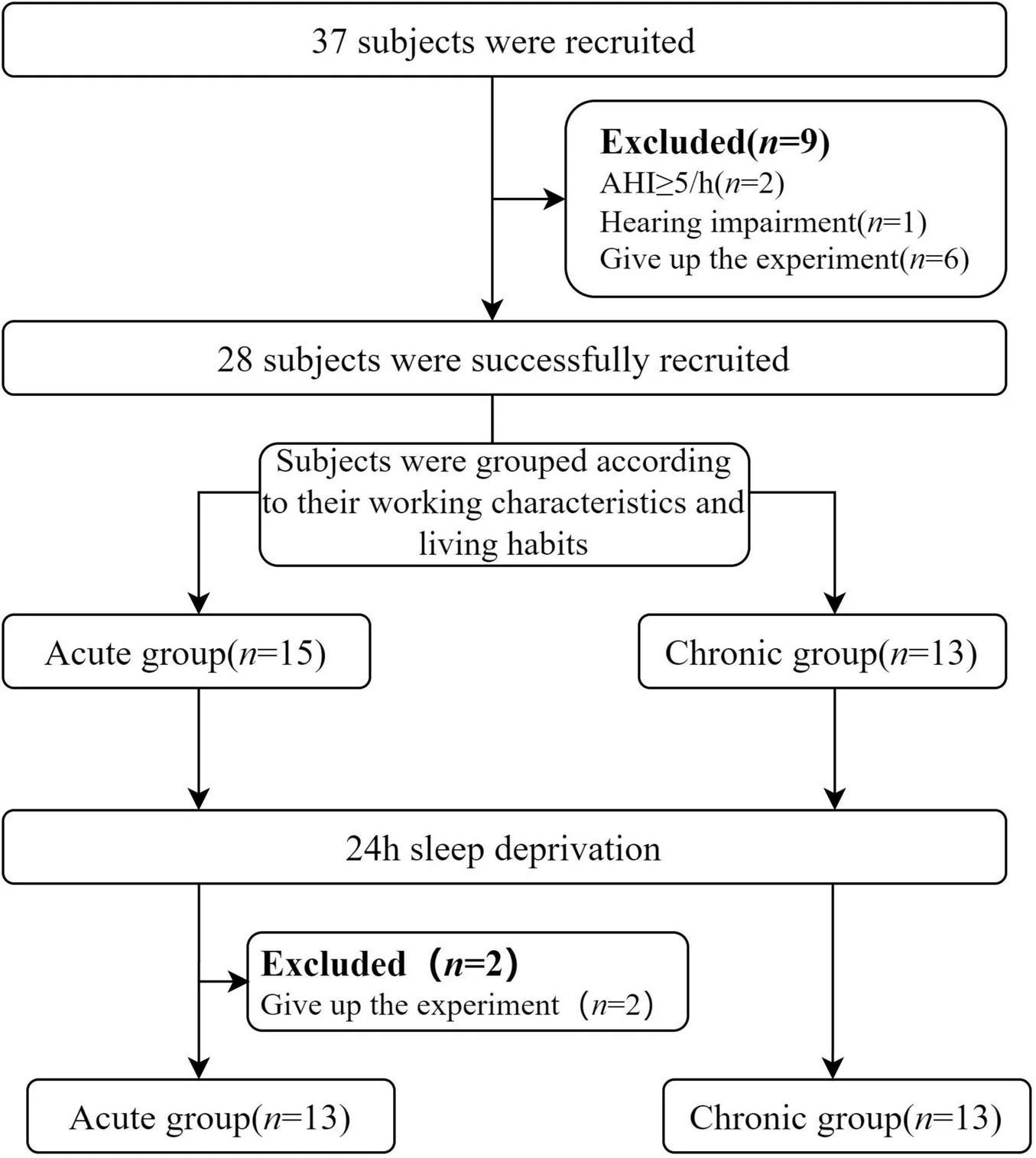
Flowchart of subjects’ recruitment and experiment. AHI, Apnea Hypopnea Index.

This research was conducted at Tianjin First Central Hospital, Tianjin, and involved recruiting healthy adult participants. To ensure the accuracy and reliability of the study, all participants underwent thorough health screening before participation, including assessments using polysomnography (PSG) conducted by two physicians experienced in sleep medicine. The exclusion criteria included (1) a history of substance abuse, including psychiatric drugs or alcohol; (2) a history of psychiatric or neurological disorders; (3) a history of head trauma; (4) a family history of mental illness; (5) hearing impairment or difficulty in discerning sounds. Additionally, detailed pharmacological histories were obtained for all participants to exclude individuals with any current or past medication use that could potentially affect sleep architecture or cognitive function. Specifically, participants using benzodiazepines (e.g., diazepam, lorazepam), non-benzodiazepine hypnotics (e.g., zolpidem), antidepressants (e.g., fluoxetine, amitriptyline), antipsychotics (e.g., quetiapine), or stimulants (e.g., modafinil, methylphenidate) were not included in the study. The acute sleep deprivation group consisted of people with regular sleep schedules and no night shifts. The chronic sleep deprivation group included individuals engaged in long-term night shift work for over 24 months who consistently remained awake during their night shifts. All participants volunteered for the study, fully understood its purpose and content, and provided informed consent. Participants were compensated for their participation upon completion of the experiment. This study has been approved by Tianjin First Central Hospital’s ethics committee (2020N114KY). The flow chart for the recruitment of subjects and the experiments is shown in Figure 1.

### 2.2 Stimulating materials

**FIGURE 2:**
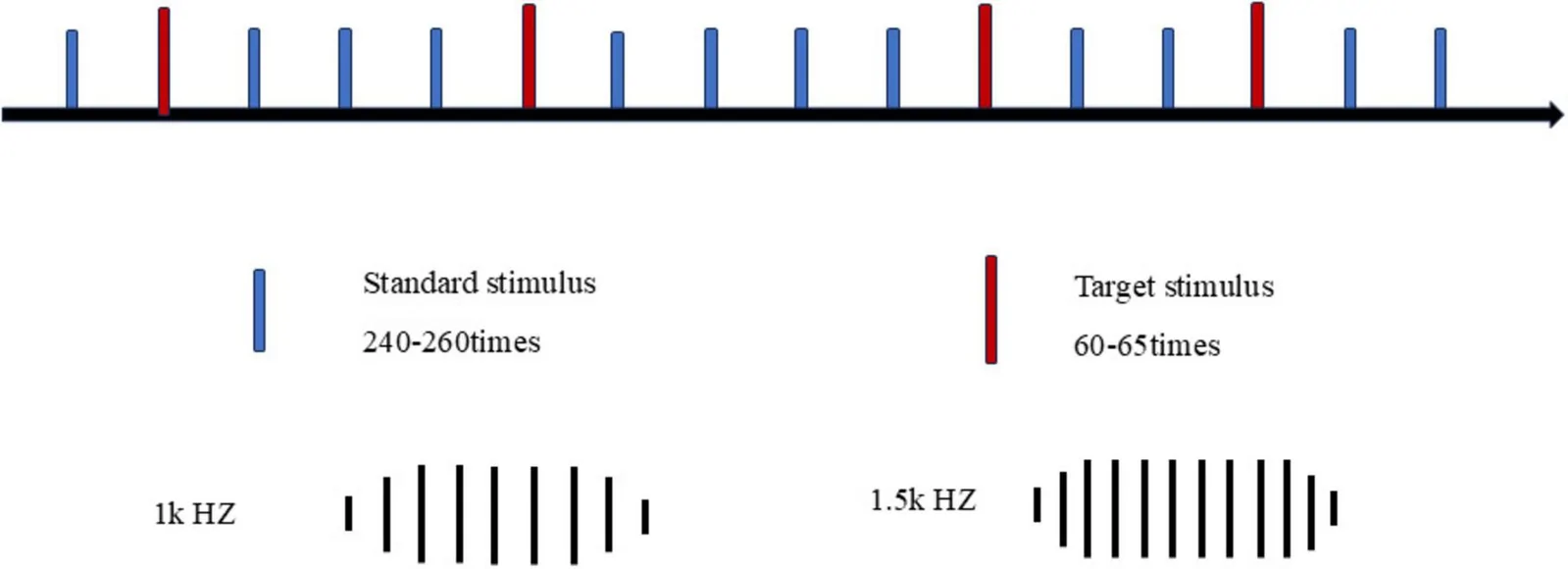
E-prime 3.0 script illustrating the auditory oddball paradigm. Blue indicates the standard stimulus, while red indicates the target stimulus.

The stimulus paradigm was developed using E-prime 3.0 psychology software, and the classical auditory Oddball paradigm was implemented (Sutton et al., 1965). The standard stimulus consisted of an 80% proportion of a 1.0 kHz pure tone, while the target stimulus comprised a 20% proportion of a 1.5 kHz pure tone (Figure 2). The number of target stimuli was between 60 and 65. The order in which stimuli were presented followed a pseudo-random sequence that prevented consecutive occurrences of two target stimuli. Auditory ERPs induced by the oddball paradigm have been widely used in neurocognition (Shymkiv et al., 2025; Gong et al., 2024). Therefore, in this study, we used the auditory oddball paradigm to induce P300, aiming to clarify the cognitive function differences between chronic and acute sleep-deprived people. This approach yields biomarkers suitable as therapeutic targets for clinical and neurocognitive interventions and translational research (Parker et al., 2021).

### 2.3 Experimental procedure

Brain electrical and behavioral data were collected from participants waking up on the day of the experiment (between 7:00 and 8:00 a.m.). Subsequently, participants were instructed to remain awake in a designated experimental area for 24 h. Throughout this period, participants were permitted to engage in light activities such as reading, watching movies, or playing card games. To maintain the accuracy of the experiment, high-intensity physical exercise and closed-eye rest were prohibited, and participants were not allowed to consume any beverages that could impact their nervous system, including coffee, tea, or alcohol. Two experimenters took turns monitoring the entire experimental process. After the 24-h sleep deprivation period, participants underwent a second round of brain electrical activity and behavioral data collection.

### 2.4 Data collection procedure

The EEG data were collected in a soundproof and electromagnetically shielded environment using the EGI GES400 brain electrode system (United States) and a HydroCel 256 electrode cap soaked in potassium chloride solution for enhanced conductivity. The electrode impedance was controlled within 50 KΩ during the data collection process. Brain electrical activity was recorded with Net Station Acquisition 5.4.3-R software, utilizing Cz as the reference electrode, a sampling rate of 1 kHz, and a filtering range of 0.1-30 Hz. The electrodes were mounted on an elastic cap according to the 10–20 system standard positions. During EEG acquisition, participants were instructed to fixate on a central white cross on the display screen and avoid excessive facial, eye, and neck movement. They were presented with two different 70 dB sounds: a standard low-frequency sound wave at 1 kHz and a target high-frequency sound wave at 1.5 kHz. When hearing the high-frequency sound wave, participants were required to respond by pressing a button as quickly as possible while mentally counting their presses. The response box recorded the time from sound emission to the participant’s response and determined whether they made the correct judgment.

### 2.5 EEG data analysis

All EEG data collected in this study were analyzed and visualized using MATLAB R2023b software, using the EEGLAB2021_0 toolbox (Delorme and Makeig, 2004). The data preprocessing steps included: (1) Implementing filters such as high-pass filtering at 1 Hz, low-pass filtering at 30 Hz, and notch filtering at 50 Hz to reduce electrode drift, muscle noise, and power line interference; (2) Downsampling to 250 Hz; (3) Interpolating signals for affected electrodes and manually removing bad segments; (4) Re-referencing to the average of all electrodes (Hu et al., 2017); (5) Segmenting the data from –200 ms before to 800 ms after the stimulus marker, with baseline correction based on the –200 ms preceding the stimulus; (6) Applying Independent Component Analysis (ICA) to remove artifacts related to blinking, saccades, ECG, and muscle activity (Mognon et al., 2011); (7) Excluding extreme values beyond ± 100 μV; (8) The P300 component is usually the most prominent in the parietal electrode with a peak latency of approximately 300–600 ms (Parker et al., 2021). Thus, the average mean P300 latency and amplitude with a latency of 300–600 ms was extracted across the nine electrodes surrounding the Cz electrode in this study (Friedman et al., 2001). The P300 components were extracted and quantified using the ERPLAB v12.00 plugin (Lopez-Calderon and Luck, 2014). Special attention was given during this process to exclude poor-quality data or those with excessive interference due to participant cooperation issues or other factors. Only reaction time data from participants who correctly responded to the target stimulus were included for statistical analysis, while any incorrect responses were excluded.

### 2.6 Statistical methods

Statistical analyses were conducted using SPSS 25.0 software. Quantitative data following normal distribution were presented as $\bar{\text{x}}$ ± SD. Pre- and post-experiment data were compared using a paired samples *t*-test. Repeated-measures analysis of variance (RM-ANOVA) was performed to examine the effects of sleep condition and the duration of night shifts on reaction time and P300. The within-subject factor was sleep condition, and the between-subject factor was the duration of night shifts. The model included the main effects of the duration of night shifts, sleep condition, and the interaction effect. Partial eta squared (η_*p*_^2^) was reported as a measure of effect size. Pairwise comparisons were conducted using Bonferroni correction when significant effects were observed. A significance level of *P* < 0.05 was applied. Linear regression and correlation analyses were performed using GraphPad Prism 9.0 software, with statistical significance set at *P* < 0.05.

## 3 Results

### 3.1 Night shift characteristics of subjects

Table 1 shows the duration of night shift work and the frequency of night shifts per month for both groups. A total of 26 participants met the inclusion criteria. Based on the duration of night shifts, they were categorized into two groups: an acute sleep deprivation group (defined as no night shift work, *n* = 13) and a chronic sleep deprivation group (defined as more than 24 months of night shift work, *n* = 13).

**TABLE 1:** Describe the night shift characteristics of the two groups.

	Acute group (*n* = 13)	Chronic group (*n* = 13)
Age: $\bar{x}$ ± S, years	29.31 ± 4.17	30.23 ± 3.77
Gender: n (%)		
Man	8 (61.54)	4 (30.77)
Woman	5 (38.46)	9 (69.23)
Night shift duration: *M* (*Q*_1_, *Q*_3_), months	No	49.00 (30.50, 86.50)
Number of night shifts per month: *M* (*Q*_1_, *Q*_3_), times	No	6.00 (5.50, 7.00)

### 3.2 Behavioral results

**FIGURE 3:**
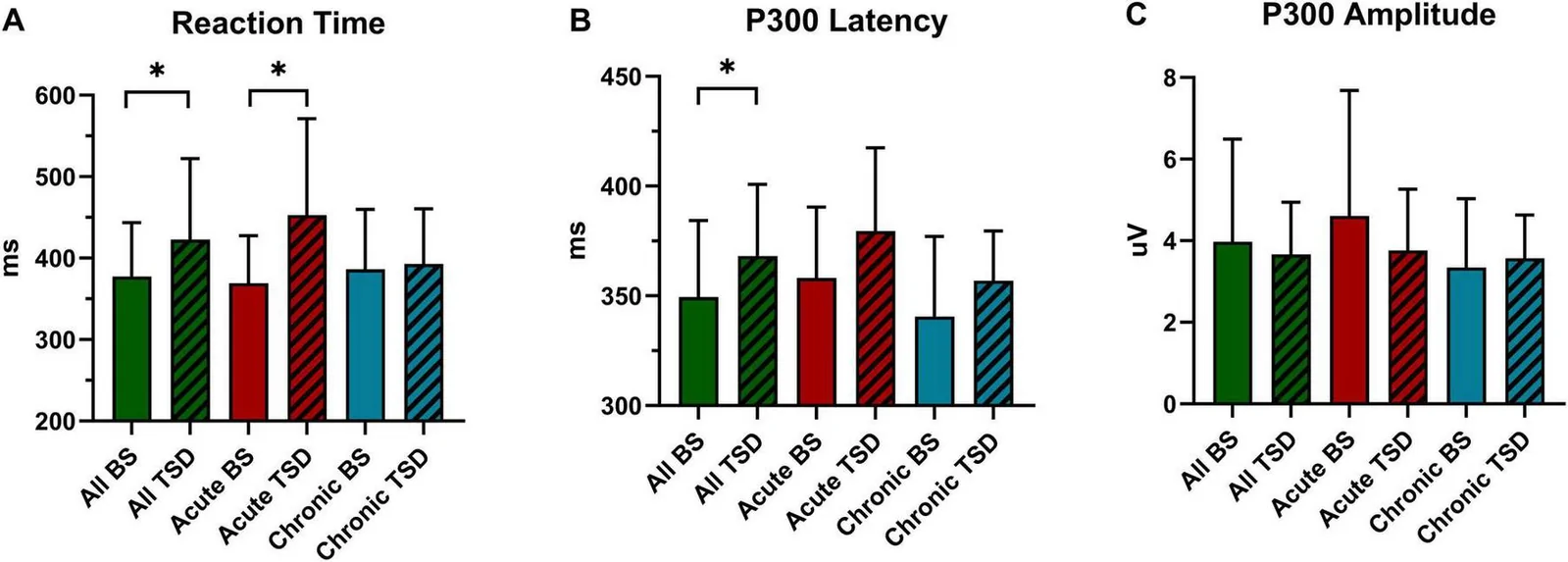
Inter-group and intra-group statistical analysis of reaction time and P300 before and after sleep deprivation. **(A)** Reaction Time. **(B)** P300 Latency. **(C)** P300 Amplitude. All, 26 subjects. BS, at baseline; TSD, after total sleep deprivation. *P < 0.05.

The results of reaction time under two different sleep condition are presented in Table 2 and Figure 3A. A paired-sample *t*-test conducted before and after the sleep deprivation experiment revealed a significant prolongation in reaction time for all participants following the experiment (*t* = −2.381, *P* = 0.025). Specifically, the reaction time of the acute group was significantly prolonged by 83.69 ms (*F* = 11.179, *P* = 0.003). In contrast, the chronic group exhibited only a minor increase in reaction time of 6.54 ms (*F* = 0.071, *P* = 0.793). RM-ANOVA was performed to examine the effects of sleep condition on reaction time in chronic and acute groups. The main effect of the night shift history duration was not significant [*F_(*a*)_* = 0.615, *P* = 0.440, η_*p*_^2^ = 0.025]. However, the main effect of sleep condition was significant [*F_(*b*)_* = 6.514, *P* = 0.017, η_*p*_^2^ = 0.213], suggesting that reaction time was significantly altered following sleep deprivation. Additionally, a significant interaction effect between the night shift history duration and sleep condition was observed [*F_(*a*X__*b*)_* = 4.736, *P* = 0.040, η_*p*_^2^ = 0.165], indicating that the impact of sleep deprivation on reaction time differed between the chronic and acute groups.

**TABLE 2:** Means and standard deviations of reaction time(ms) characteristics.

	Chronic group	Acute group	^1^Difference value
BS	386.15 ± 73.83	369.08 ± 58.56	17.07
TSD	392.69 ± 67.81	452.77 ± 118.26	−60.08
^2^Difference value	6.54	83.69	
RM-ANOVA			
*F_(*a)*_*	*F* = 0.615, *P* = 0.440, η*_*p*_*^2^ = 0.025		
*F_(*b)*_*	*F* = 6.514, *P* = 0.017*, η*_*p*_*^2^ = 0.213		
*F_(*a*__X__*b)*_*	*F* = 4.736, *P* = 0.040*, η*_*p*_*^2^ = 0.165		

BS, at baseline; TSD, after total sleep deprivation.

^1^Difference value, Chronic group – Acute group.

^2^Difference value, TSD – BS; *F*_(a)_, *F*_(the duration of night shifts)_; *F*_(b)_, *F*_(sleep condition)_; *F*_(a__X__b)_, *F*_(the duration of night shifts Xsleep condition)._ *P < 0.05.

### 3.3 ERPs results

**FIGURE 4:**
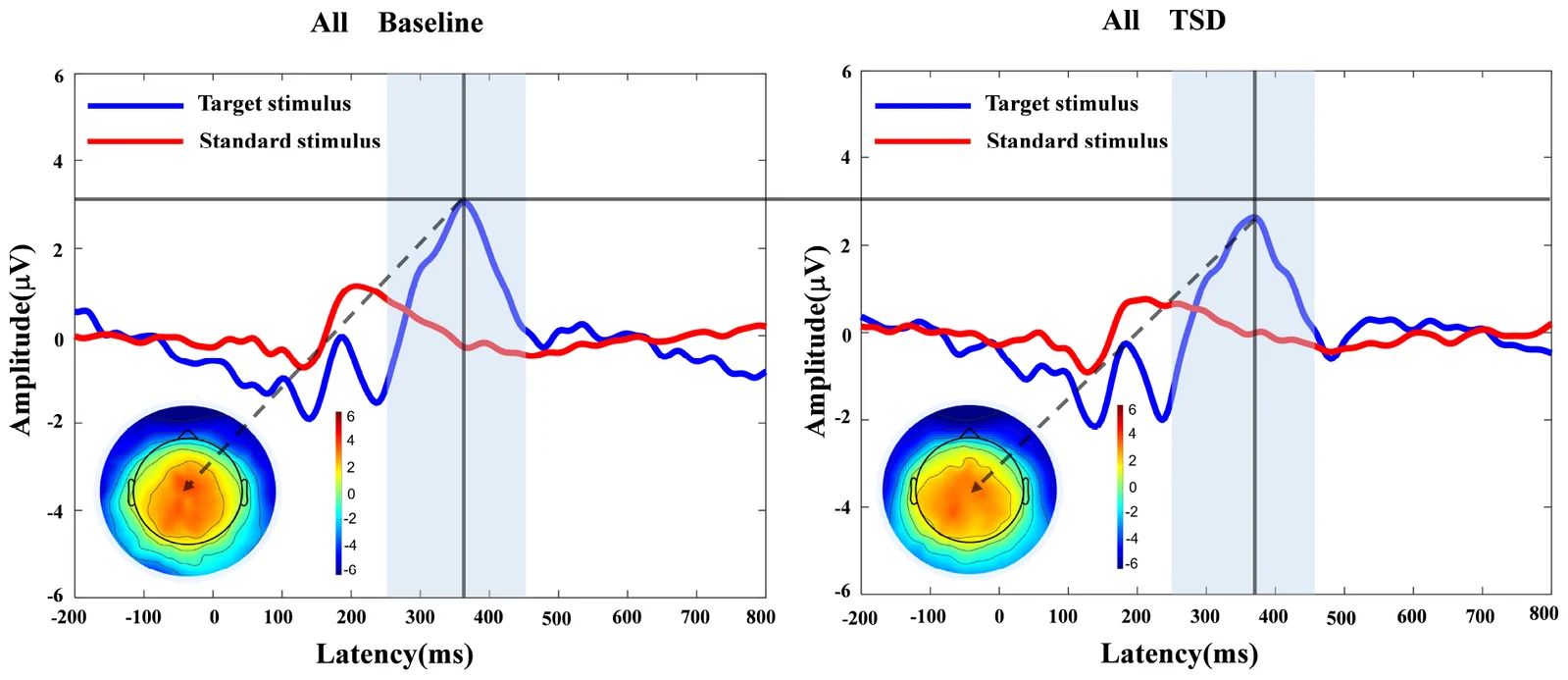
MATLAB was utilized to analyze the P300 component of 26 participants before and after sleep deprivation. Grand average of the P300 (250–450 ms) latency and amplitude induced by the target stimulus of the auditory oddball. The average of the nine electrodes surrounding the Cz electrode represented the P300 component. The results indicated a prolongation in P300 latency and a reduction in P300 amplitude after sleep deprivation compared to baseline. The terrain distribution represented by the color topographic map is extracted at the time point with the largest amplitude. All, 26 subjects; BS, at baseline; TSD, after total sleep deprivation.

Tables 3, 4 and Figures 3B,C, 4 present the result of P300 latency and amplitude in both groups at baseline and after TSD.

**TABLE 3:** Means and standard deviations of P300 latency(ms) characteristics.

	Chronic group	Acute group	^1^Difference value
BS	340.62 ± 36.43	358.15 ± 32.23	−17.53
TSD	356.92 ± 22.58	379.38 ± 38.01	−22.46
^2^Difference value	16.3	21.23	
RM-ANOVA			
*F_(*a)*_*	*F* = 3.906, *P* = 0.060, η_p_^2^ = 0.140		
*F_(*b)*_*	*F* = 5.523, *P* = 0.027*, η*_*p*_*^2^ = 0.187		
*F_(*a*__X__b)_*	*F* = 0.095, *P* = 0.761, η*_*p*_*^2^ = 0.004		

BS, at baseline; TSD, after total sleep deprivation.

^1^Difference value, Chronic group – Acute group.

^2^Difference value, TSD – BS; F_(*a)*_, F_(*the duration of night shifts)*_; F_(*b*)_, F_(*sleep condition)*_; F_(*a*__X__ b)_, F_(*the duration of night shifts*_ _Xsleep condition)._*, *P* < 0.05.

**TABLE 4:** Means and standard deviations of P300 amplitude (μV) characteristics.

	Chronic group	Acute group	^1^Difference value
BS	3.34	4.61	−1.27
TSD	3.57	3.76	−1.19
^2^Difference value	0.23	−0.85	
RM-ANOVA			
*F_(*a)*_*	*F* = 1.138, *P* = 0.297, η*_*p*_*^2^ = 0.045		
*F_(*b)*_*	*F* = 0.709, *P* = 0.408, η*_*p*_*^2^ = 0.029		
*F_(*a*__X b)_*	*F* = 2.145, *P* = 0.156, η*_*p*_*^2^ = 0.082		

BS, at baseline; TSD, after total sleep deprivation.

^1^Difference value, Chronic group – Acute group.

^2^Difference value, TSD – BS; *F*_(a)_, *F*_(the duration of night shifts)_; *F*_(b)_, *F*_(sleep condition)_; *F*_(a__X__b)_, *F*_(the duration of night shifts Xsleep condition)._ *P < 0.05.

#### 3.3.1 P300 Latency

A paired-sample *t*-test conducted before and after the sleep deprivation experiment revealed that the P300 latency was significantly extended by 18.76 ms (*t* = 2.394, *P* = 0.024). RM-ANOVA was conducted to evaluate the effects of sleep condition on P300 latency in the chronic and acute groups. There was slight statistical significance in the main effect of night shift history duration [*F_(*a*)_* = 3.906, *P* = 0.060, η*_*p*_*^2^ = 0.140]. Further research to expand the sample size may yield surprising results. However, a significant main effect of sleep condition was observed [*F_(*b*)_* = 5.523, *P* = 0.027, η*_*p*_*^2^ = 0.187], indicating that the impact of sleep deprivation significantly influenced P300 latency. There is no significant interaction effect was found [*F_(*a*X*b*)_* = 0.095, *P* = 0.761, η*_*p*_*^2^ = 0.004], suggesting that the differences in P300 latency in the two groups were equally distributed across sleep conditions.

#### 3.3.2 P300 amplitude

The P300 amplitude of all participants following the sleep deprivation experiment decreased by 0.30 μV (*t* = 0.803, *P* = 0.418). Specifically, The P300 amplitude in the acute group decreased by 0.85 μV (*F* = 2.660, *P* = 0.116), while it increased by 0.23μV (*F* = 0.194, *P* = 0.664) in the chronic group. The main effect of the night shift duration was not significant [*F_(*a*)_* = 1.138, *P* = 0.297, η*_*p*_*^2^ = 0.045]. Similarly, the main effect of sleep condition was not significant [*F_(*b*)_* = 0.709, *P* = 0.408, η*_*p*_*^2^ = 0.029]. Although the main effect of the night shift duration and sleep condition on P300 amplitude did not reach statistical significance, the potential effect trend can be observed in Figure 3C. Additionally, the interaction effect failed to reach significance [*F_(*a*X*b*)_* = 2.145, *P* = 0.156, η*_*p*_*^2^ = 0.082], indicating that no significant interaction effect between night shift duration and sleep deprivation was observed for P300 amplitude. Given the significant changes in P300 latency and reaction time, the non-significant amplitude findings do not necessarily indicate the absence of an effect but rather suggest that the impact may be more subtle or influenced by additional factors. Future research with a larger sample and refined methodological controls may help clarify this relationship.

### 3.4 Correlation analysis

**FIGURE 5:**
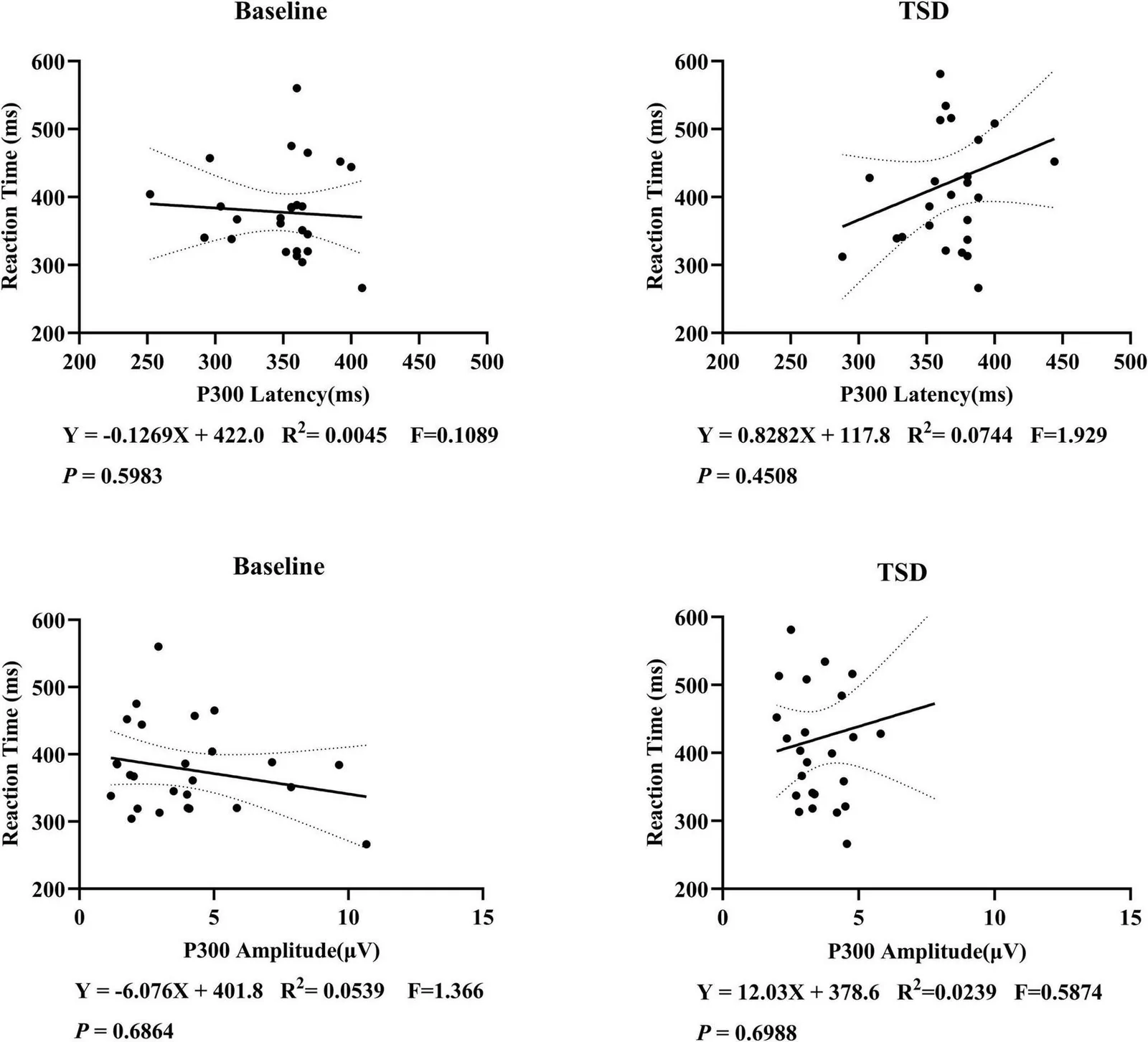
Correlation analysis of P300 latency, amplitude, and reaction time in 26 subjects. A linear correlation between P300 and subjective reaction time has not been established.

**FIGURE 6:**
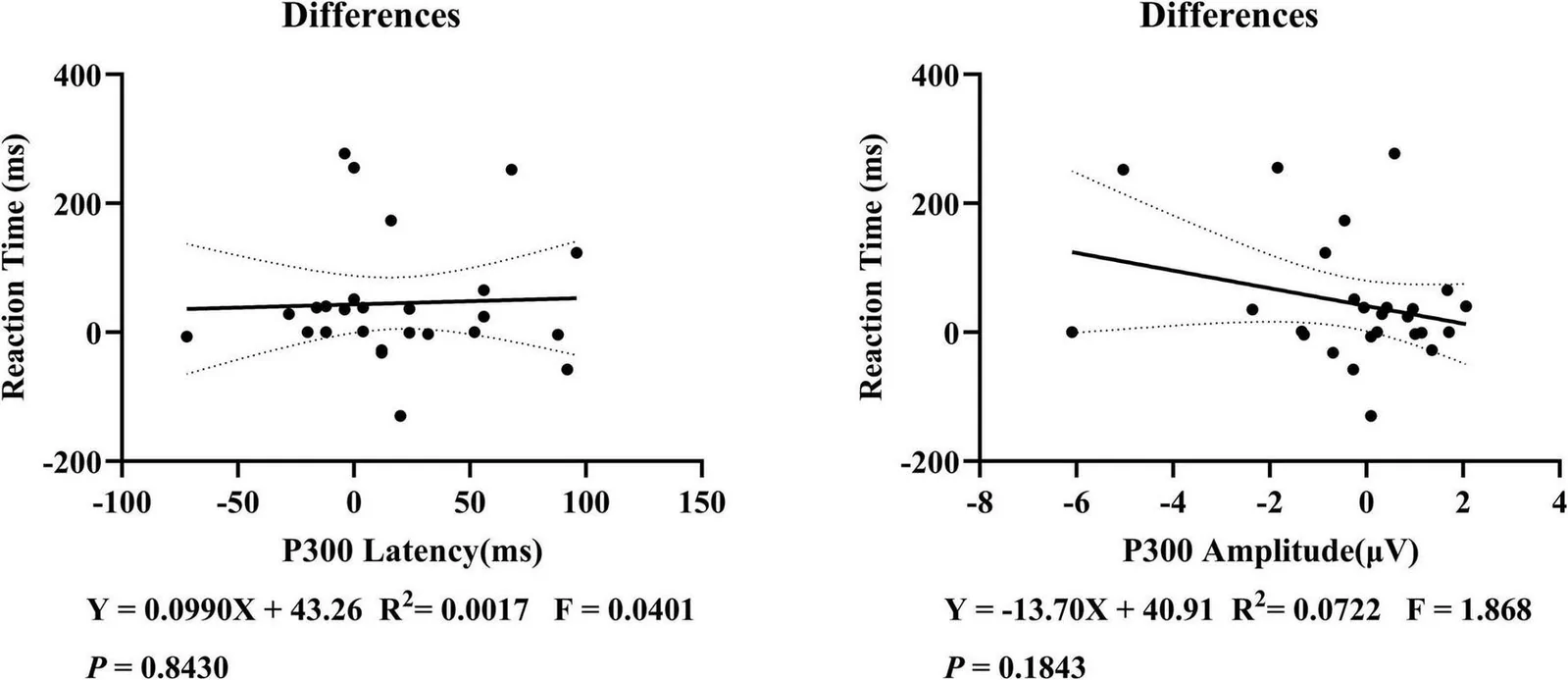
Correlation analysis of the changes in P300 latency, amplitude, and reaction time before and after the sleep deprivation experiment in 26 subjects. On further examination, a linear correlation between P300 and subjective reaction time was still not found in the changes before and after sleep deprivation.

This study examined the correlation between P300 latency and amplitude with subjective reaction time, but no significant linear correlation was found (Figure 5). Further correlation analyses were conducted on the changes before and after sleep deprivation. It was observed that there was a marginal statistical significance in the decrease of P300 amplitude as reaction time increased (Figure 6).

## 4 Discussion

This study unveiled a significant increase in P300 latency and reaction time among participants following a 24-h sleep deprivation experiment. Interestingly, individuals who frequently work night shifts exhibited less cognitive impairment after the sleep deprivation experiment. In contrast, those with no night shifts showed a notable decline in cognitive function, particularly regarding reaction ability. While individuals in the chronic group demonstrated greater tolerance for sleep deprivation, they experienced chronic cognitive impairments as a result of frequent night shifts. The impact of sleep deprivation on the P300 component and reaction time did not exhibit synchronized degrees of effect.

Our study found that sleep deprivation can lead to prolonged P300 latency, a slight decrease in amplitude, and increased reaction time. Sleep deprivation can reduce attention, alertness, and reaction capability. The prolonged P300 latency may be associated with decreased sensitivity of brainstem neurons to auditory signals, a reduction in signals received by the auditory cortex, and a slower cognitive processing speed (Hayashi et al., 2005). The decreased P300 amplitude indicates a decline in selective attention and memory functions. P300 amplitude represents the strength of EEG potential and is closely related to the number, activity, and direction of neurons involved in synchronous firing. Previous studies have shown that prolonged sleep deprivation exceeding 24 h can disrupt hippocampal cell proliferation, differentiation, and survival (Mueller et al., 2015). Sleep is essential for memory consolidation, recovery, and cognitive function optimization (Liberalesso et al., 2012; Herculano-Houzel, 2013). The present study’s subjective and objective assessments indicated that 24-h sleep deprivation resulted in neurological dysfunction and impaired cognitive function. Adequate sleep is essential for overall health, learning, and wellness.

Our research revealed that the chronic group exhibited a 17.07 ms longer reaction time than the acute group before the sleep deprivation experiment and a 1.27 μV lower P300 amplitude. This suggests potential chronic cognitive impairment in their daily lives, possibly linked to chronic neuroinflammation. Choshen-Hillel et al. (2021) found that residents or attending physicians working 4-8 night shifts per month for 4 consecutive years experienced reduced cortisol levels and increased inflammatory factors in the morning, leading to decreased executive function, slower brain processing speed, and heightened impulsivity. After completing a night shift, during which they remained awake all night, resident physicians continued to work. Their brain’s ability to encode memory information decreased, leading to a higher likelihood of forming incorrect memories (Frenda et al., 2014), resulting in a 20% increase in error rate and a 14% longer time required to complete tasks (Taffinder et al., 1998). Studies have indicated that long-term shift workers are at an increased risk of drowsiness while driving, with an accident rate 2.3 times higher than non-shift workers (Barger et al., 2005). Chronic sleep deprivation specifically impacted hippocampal ripples that support memory formation, weakening their efficacy and causing damage to brain memory function (Giri et al., 2024). The effects of sleep deprivation cannot be fully restored. Individuals with a history of frequent night shift work may show cognitive impairment even when they get sufficient sleep. This is a reminder that staying up late can lead to irreversible damage to neuronal cells, which cannot be fully compensated for by additional sleep. Cognitive impairment has a cumulative effect, with more severe impairment observed in those who have worked night shifts for more extended periods or more frequently.

Our research indicates that long-term frequent night shift workers have developed a heightened tolerance for sleep deprivation. Following a night shift, they may experience a brief period of increased mental arousal, demonstrating heightened alertness, attention, reaction ability, and judgment. The body is suspected to have developed self-protective mechanisms in response to prolonged damage to neural cells. Long-term frequent night shifts may also have altered circadian rhythms, brain metabolic stability, or even gene expression in these individuals. Andreadou et al. (2023) discovered that IL-12 could mitigate neuroinflammation and has direct neuroprotective and neurotrophic effects on neurons. IL-12 is expressed in mouse and human neural ectoderm and can prevent early neurodegenerative changes. Neurons can release neurotrophic factors to counteract central nervous system damage in various brain diseases (Jin et al., 2005). Chronic sleep deprivation can lead to abnormal expression of clock genes (Niu et al., 2022), disrupting the body’s circadian rhythm (Franken and Dijk, 2009). Long-term night shift work can further alter circadian rhythms due to nighttime light exposure and changes in food intake, ultimately reshaping the biological clocks of individuals. The mechanisms involved in this process require further investigation. The results of this study confirm that long-term frequent night shift workers have developed a high tolerance for sleep deprivation, which may be related to the reshaping of their biological clocks and the formation of self-protection mechanisms.

Our study performed a correlation analysis of the P300 component and reaction time. No linear correlation was found. However, this finding raises further questions regarding the potential existence of an unresolved correlation between the objective P300 component and subjective reaction time. P300 is widely recognized as an objective indicator of neural and cognitive function (Demirayak et al., 2023). It serves as a reflection of how the brain allocates attention and processes target information during mental tasks. The characteristics of P300—such as its latency and amplitude—indicate neuronal activity levels. Importantly, these neural responses occur independently from conscious control or awareness (Nani et al., 2019). In contrast, reaction time in this study represents another facet of cognitive function that necessitates conscious control by individuals when responding to stimuli. Specifically, responding to auditory signals involves complex neurophysiological mechanisms, including sensory processing pathways within the central nervous system. This process encompasses various stages such as signal detection, transmission through neural circuits, integration within cortical areas responsible for decision-making, and motor response execution. Combined with the distribution of scatter plot data, we found that sleep deprivation had a slightly greater effect on the P300 component, and we speculate that EEG data are more sensitive than reaction time data in reflecting changes in cognitive function.

Due to the difficulty of the 24-h sleep deprivation experiment, only a limited number of participants were able to complete the study with high-quality data. While our findings provide valuable insights, the relatively small sample size may limit the generalizability of the results. Therefore, further research with a larger cohort is warranted to validate these findings. Despite observing marginal statistical significance, we cannot definitively conclude an association between P300 components and reaction time. The error rate in behavioral experiments for all participants was low, with fewer than five errors made across 300 judgments and over half of the chronic group exhibiting zero errors. We employed the auditory Oddball paradigm in this experiment, which was considered to have a simple task difficulty. In future studies, task difficulty could be increased, or a more suitable paradigm could be considered.

## 5 Conclusion

In conclusion, based on the P300 component and the reaction time, our study demonstrates that sleep deprivation could significantly impact cognitive function. This study also indicates that people who work regular night shifts are at risk for chronic cognitive impairment. Workers experiencing long-term circadian rhythm reversal cannot escape the cumulative effects of cognitive impairment, even with adequate sleep. Sleep deprivation adversely impacts both the objective P300 component and subjective reaction time, although the extent of these impairments does not occur synchronously. Paying attention to cognitive impairments following night shifts is imperative to reduce the incidence of decreased industrial productivity and traffic accidents.

## Data Availability

The raw data supporting the conclusions of this article will be made available by the authors, without undue reservation.
