# Correction: The impact of sleep deprivation on cognitive function in healthy adults: insights from auditory P300 and reaction time analysis

**DOI:** 10.3389/fnins.2026.2023176

**Published:** 2026-10-27

**Authors:** Zhongkai Ren, Xiang Mao, Ziyue Zhang, Wei Wang

**Affiliations:** 1 Department of Otorhinolaryngology-Head and Neck Surgery, Tianjin First Central Hospital, Tianjin, China; 2 Institute of Otolaryngology of Tianjin, Tianjin, China; 3 Key Laboratory of Auditory Speech and Balance Medicine, Tianjin, China; 4 Key Medical Discipline of Tianjin (Otolaryngology), Tianjin, China; 5 Otolaryngology Clinical Quality Control Centre, Tianjin, China; 6 First Central Clinical College, Tianjin Medical University, Tianjin, China

**Keywords:** sleep deprivation, EEG, event-related potentials, P300, cognition

The parenthetical statement “(*P* < 0.05)” was inadvertently included. The correct statistical result is *P* = 0.793, as reported in Section 3.2.

A correction has been made to the **Results** section of the **Abstract**:

“In contrast, the reaction time in the chronic group exhibited only a minor increase of 6.54 ms.”

There was a mistake in Table 1 as published. In the row describing night-shift duration, the word “mouths” was a typographical error and should read “months.” The corrected Table 1 appears below.

**Table 1:** Describe the night shift characteristics of the two groups.

Characteristics	Acute group (*n* = 13)	Chronic group (*n* = 13)
Age: $\bar{\text{x}}$ ± S, years	29.31 ± 4.17	30.23 ± 3.77
Gender: *n* (%)		
Man	8 (61.54)	4 (30.77)
Woman	5 (38.46)	9 (69.23)
Night shift duration: *M* (*Q_1_, Q_3_*), months	No	49.00 (30.50, 86.50)
Number of night shifts per month: *M* (*Q_1_, Q_3_*), times	No	6.00 (5.50, 7.00)

The leading “−2” in the reported t value was inadvertently omitted during transcription. The *P* value remains unchanged.

A correction has been made to Section 3.2, **Behavioral results**, first paragraph:

“A paired-sample *t*-test conducted before and after the sleep deprivation experiment revealed a significant prolongation in reaction time for all participants following the experiment (*t* = −2.381, *P* = 0.025).”

There was a mistake in Figure 4 as published. The positive and negative signs on the *y*-axes of both panels were inadvertently reversed, and the unit “uV” should read “μV.” In the corrected Figure 4, the *y*-axes range from +6 μV at the top to −6 μV at the bottom. This was a figure-labeling error and does not affect the underlying data. The corrected Figure 4 appears below.

**Figure 4:**
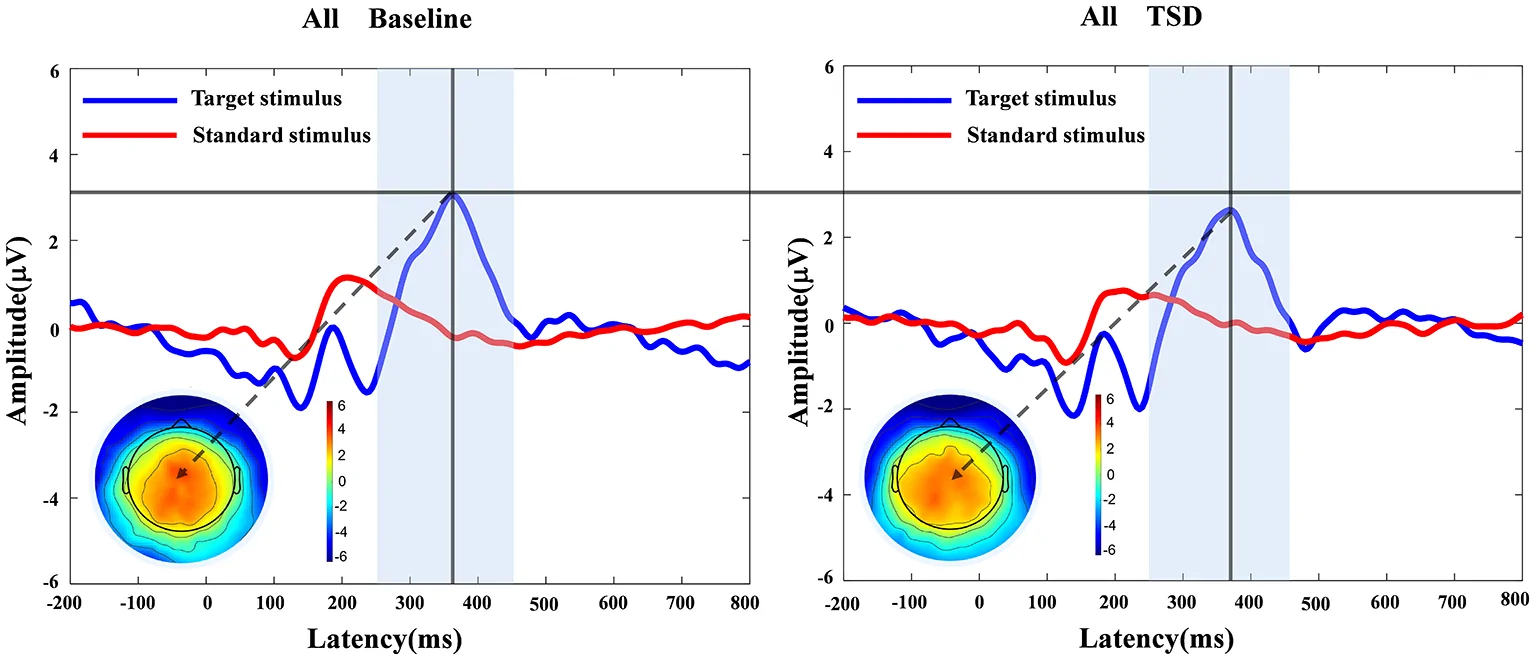
MATLAB was utilized to analyze the P300 component of 26 participants before and after sleep deprivation. Grand average of the P300 (250–450 ms) latency and amplitude induced by the target stimulus of the auditory oddball. The average of the nine electrodes surrounding the Cz electrode represented the P300 component. The results indicated a prolongation in P300 latency and a reduction in P300 amplitude after sleep deprivation compared to baseline. The terrain distribution represented by the color topographic map is extracted at the time point with the largest amplitude. All, 26 subjects; BS, at baseline; TSD, after total sleep deprivation.

The original version of this article has been updated.

